# Comparing laboratory and online settings: equivalence in training and transfer effects for training task-order coordination processes

**DOI:** 10.3389/fpsyg.2024.1440057

**Published:** 2024-10-01

**Authors:** Daniel A. Darnstaedt, Leif Langsdorf, Torsten Schubert

**Affiliations:** Department of Psychology, Martin-Luther University Halle-Wittenberg, Halle (Saale), Germany

**Keywords:** cognitive training, task-order coordination, stimulus-unspecific transfer, online-lab comparison, dual task training

## Abstract

**Introduction:**

The literature on dual-task training suggests reductions in task-coordination costs with extensive practice, yet such regimens are resource-intensive. This study investigates the feasibility of online assessments for cognitive training studies by comparing training and transfer effects on task-*order* coordination (TOC) skills in laboratory versus online settings.

**Methods:**

We conducted a 5-day training regimen including pre-and post-test. Sixty-two participants completed training either in our laboratory or online via Pavlovia. They were assigned to one of two training order conditions, either practicing two visual-manual tasks in a dual-task situation with fixed task order or with random task order. Performance metrics included reaction time (RT) and error rates for trained and untrained tasks to assess TOC costs before and after the training. Data from both setting conditions (laboratory vs. online) were compared.

**Results:**

Firstly, data of both settings revealed training-order specific training and transfer effects for TOC costs on RT level. Random task order training improved TOC for trained and untrained tasks, whereas fixed order training did not. Secondly, cross-setting analyses, both frequentists and Bayesian, confirmed these effects and revealed no reliable impact of setting on outcomes.

**Discussion:**

This research carries two significant implications. Our findings demonstrate the acquisition of task-*order* coordination skills, extending prior research on improving task-coordination in dual-task situations. Additionally, the robust effects for such improvements were independent of specific tasks and setting (whether investigated online or in the laboratory), supporting the use of online testing in cognitive training regimens for resource savings without compromising quality.

## 1 Introduction

Performing two component tasks at the same time, known as dual tasks (DT), can be quite challenging in unpracticed situations. In these situations, performance of one or both tasks is usually impaired, thus leading to DT costs. The DT costs are reflected in slower reaction times (RTs) and/or increased error rates in contrast to single task situations (Pashler, 1994; Schubert et al., 2008; Welford, 1952), but it has been shown that one can reduce these costs with extensive practice. For example, when learning a musical instrument, at the beginning, novices struggle to coordinate various task aspects such as finger placement, rhythm, and reading the note sheets. However, as people continue to practice, they gradually become capable of coordinating these different aspects, which allows them to play the instrument more proficiently. The improved coordination of multiple task requirements is a result of ongoing practice and can lead to enhanced performance when conducting various activities while playing the instrument.

While this example demonstrates a common everyday situation in which practice can improve the coordination of multiple tasks, in the psychological laboratory rather simple and well-controlled component tasks, such as choice reaction-time tasks are used to investigate the regularities of DT processing and its training-related improvement. In order to examine the training-related acquisition of task-coordination skills, rigorous training protocols have been employed, which require participants to undergo several laboratory sessions across multiple days (e.g., Liepelt et al., 2011; Schubert et al., 2017; Schubert and Strobach, 2018; Strobach et al., 2014). Of course, such an experimental procedure of conducting multiple-day training is time-consuming and it requires a high level of commitment from the participants and of experimental effort as well. Therefore, alternative approaches are highly welcome, which allow researchers to optimize the large demand of time, labor resources, and even economical costs that are related to such long-lasting training procedures.

Therefore, the primary objective of the current study was to assess the potentials of online training regimens for studies focusing on training-related improvements of task-coordination, by comparing it with effects obtained in a traditional laboratory-based training. That in turn could provide valuable evidence supporting the feasibility of online training studies as a means to examine the acquisition of task-coordination skills.

Next, we will describe what is known from laboratory training studies about the cognitive processes and mechanisms of task-coordination skills. This will allow us to form assumptions about which processes can or cannot be enhanced through laboratory and online training procedures. Subsequently, we will specify the characteristics of laboratory and online training approaches and then report the findings of an empirical investigation, which compared training and transfer effects on task coordination after laboratory and online training.

### 1.1 Training-related changes in task coordination

Evidence for the acquisition of task coordination stems from studies comparing participants’ DT performance following training in DT situations versus single-task (ST) situations (Hirst et al., 1980; Liepelt et al., 2011; Schubert et al., 2017; Schubert and Strobach, 2018; Strobach et al., 2015). Practice with isolated component tasks, i.e., in ST situations, can enhance DT performance, which is often attributed to task automatization (Logan, 1988; Maquestiaux et al., 2008; Schneider and Shiffrin, 1977) and/or stage shortening (Ruthruff et al., 2006b; Strobach et al., 2013; Strobach and Schubert, 2017b). However, further advantages are observed when practicing these tasks concurrently in DT situations (but see Hartley et al., 2011; Ruthruff et al., 2006a; Schumacher et al., 2001, for a different approach combining ST and DT trials during training to boost DT performance).

This improvement is thought to result from enhanced task-coordination skills developed during DT training, which involves repeated bottleneck processing. In contrast, ST training lacks this simultaneous task processing, limiting coordination improvements (Liepelt et al., 2011; Schubert et al., 2017; Schubert and Strobach, 2018; see also Strobach, 2020; Strobach and Schubert, 2017a for recent reviews). Specifically, the authors interrupt the two task chains at the central processing stages and causes their sequential processing (Pashler, 1994; Schubert, 1999). Consequently, task coordination improvements are exclusive to DT training, as ST training fails to engage in repeated bottleneck processing (e.g., Liepelt et al., 2011; Schubert et al., 2017).

Interestingly, these studies demonstrate that improvements in task coordination extend beyond the trained tasks to novel DT situations, indicating independence from specific stimuli and stimulus–response mappings (Kramer et al., 1995; Liepelt et al., 2011; Schubert et al., 2017; Strobach and Schubert, 2017a).

Importantly, efficient task coordination requires not only the rapid switching between two task streams but the regulation of temporal order of tasks as had recently been proposed by studies highlighting that task-order coordination (TOC) is an important executive mechanism in DT situations involving a bottleneck (Luria and Meiran, 2003, 2006; Schubert, 1999; Schubert et al., 2008; Szameitat et al., 2006). Since a bottleneck requires sequential processing of the two tasks, several accounts have proposed that bottleneck processing is actively regulated by executive control processes, which are involved in the temporal scheduling of the tasks (Kübler et al., 2022a,b). The operation of such TOC processes can be shown by comparing DT performance in task situations in which the two component tasks are presented in fixed order throughout the whole block, with the performance in situations in which the order of the two tasks varies randomly from trial to trial, so called random-order blocks (De Jong, 1995; Kübler et al., 2018; Stelzel et al., 2008; Szameitat et al., 2002). In these studies, participants are instructed to respond to both stimuli based on their sequential presentation order and the RTs and error rates are usually larger in random-order compared to fixed-order blocks. This pattern is indicative for the operation of additional control processes necessary to coordinate the processing order of both tasks in the random-order blocks. While a number of studies have provided sufficient evidence for the operation of TOC processes in unpracticed DT situations (Luria and Meiran, 2003, 2006; Schubert et al., 2008; Szameitat et al., 2002, 2006), it remains an open issue to which degree these executive control mechanisms can be optimized by extended DT practice. In the current study, we aim to elaborate on the potential training-related improvement of TOC in DT processing in addition to the primary research objective whether a training-related improvement of TOC can be achieved similarly in laboratory-based and online DT training situations.

### 1.2 Online vs. laboratory-based experiments

Recently, online or web-based testing of human subjects has become very popular in several research domains, including cognitive psychology, social science or economic research. The adherence to COVID-19 safety precautions resulted in sustained laboratory closures, which, in conjunction with recent technological advancements, led to an increasing tendency to conduct studies in online settings (see Grootswagers, 2020; Sauter et al., 2020 for a detailed overview of tools and methods of online testing). However, there are still unsolved issues about the usefulness and reliability of data obtained within such online settings. In particular, it is harder to achieve sufficient levels of validity, reliability, and controllability of both technical setup and environment in online compared to laboratory settings, which is particularly relevant when response time sensitive-psychological paradigms are applied. However, there have been several attempts in the past, which addressed these issues and showed that technical precision and accuracy of the measurement across diverse platforms and applications are sufficiently well and reach the standards of laboratory research (e.g., Anwyl-Irvine et al., 2021; Bridges et al., 2020; Uittenhove et al., 2023). In addition, several studies could provide evidence for the assumption that online and laboratory-based psychological experiments yielded comparable results for a range of different cognitive tasks (e.g., Crump et al., 2013; Semmelmann and Weigelt, 2017). However, so far, this was mainly shown for rather simple sensory-motor tasks such as Stroop task, Eriksen flanker tasks, basic attentional cueing tasks and others, requiring from participants the performance of a single task situation. In a recent study, Sauter et al. (2022) compared the RTs in online settings and in the laboratory, even for a more complex and demanding DT paradigm and showed similar response time effects in online and laboratory-based testing.

While these earlier studies indicate that online testing can be considered as a valid instrument for investigating various research questions with timing-sensitive sensory-motor tasks, it remains an open question to which degree online testing would also be suitable for the investigation of studies addressing training-related improvements in DT processing. Therefore, in the current study, we applied a DT paradigm and investigated to which degree a training-related DT performance improvement can reliably and validly be investigated within an online setting. In fact, the application of an online setting for training DT situations may expose unique challenges to the experimentation. For example, variances in participant performance may be larger in online testing compared to laboratory-based settings. If these increased variances persist each day of the training, they could accumulate over the course of the experiment, leading to a compounded inaccuracy that may diminish the observed effect size when assessing training outcomes. In addition, an important issue for multiple-session training experiments is the sustainment of participants’ motivation and attention throughout the testing sessions. Often, as individuals engage in training activities, their motivation tends to diminish over time, leading to reduced effort in the training situation and even to drop outs (Birk et al., 2016). Thus, maintaining a reliable level of task commitment of participants can be more challenging in online compared to laboratory settings, primarily due to the reduced intensity of contact between experimenter and participants.

Dual-task training is inherently demanding and intensive, which is essential for fostering effective learning outcomes. In this context, decreased task commitment and decreasing motivation can have a detrimental impact on the learning results, which might be in particularly problematic if training is assumed to affect the development of executive control functions. Thus, sustaining high levels of task commitment becomes paramount, especially in online settings, to ensure the training’s efficacy and the acquisition of cognitive skills, such as task-coordination skills.

On the other hand, online testing also offers benefits that, in turn, scale with multiple days of testing (see also Gagné and Franzen, 2023, for a comprehensive list of costs and benefits associated with online studies). For example, there is no need for physical appearance of participants in the laboratory during online experiments; therefore, participants and experimenters can reduce the related effort. Furthermore, online experiments allow for rapid data collection through parallel testing of multiple subjects in the web, which would result in large time savings. Additional advantages may include reduced social pressure for participants (even though for some participants, a lack of social interaction may lead to reduced motivation) and decreased equipment and research costs (Gagné and Franzen, 2023).

All of these aspects substantiate the research question of whether we can achieve comparable outcomes in an online training intervention as in the laboratory, potentially paving the way for future utilization of online assessments to investigate inquiries regarding cognitive training research. To achieve this goal, in the current study participants practiced a DT situation across several days in two different experimental settings, the laboratory and the online setting. We maintained as much consistency and parity as possible between both setting conditions. The recruitment strategy and experimental protocols remained identical, and participants completed an equal number of training sessions in both conditions. However, in the laboratory setting, participants were physically present at the research laboratory, where they received instructions and a debriefing, and carried out the experimental sessions in a controlled environment with direct personal contact during the whole session. Conversely, in the online setting, participants received instructions and a debriefing via video conferencing and conducted the sessions on their personal devices from their homes. Hence, the frequency of contact with the experimenter remained consistent in both setting conditions, with the primary difference being the mode of interaction. While in the laboratory setting, the interaction was conducted in form of in-person and face-to-face communication, the interaction was conducted through digital means in the online setting. This approach allowed us to control for the potential effects of experimenter presence, which may not significantly impact simple reaction time tasks (Hilbig, 2016), but could be more influential in situations in which sustaining motivation over multiple training sessions is crucial, as in the present study.

In one training order condition, participants practiced two tasks in DT blocks with fixed order, i.e., two component tasks were presented with constant order throughout the whole block. In the second training order condition, participants practiced them in DT blocks with random order, i.e., the presentation order of the two component tasks varied randomly from trial to trial. Based on earlier findings of enhanced improved task-coordination skills, we expected TOC costs (as a measure of task-*order* coordination skills) to be specifically reduced in participants who practiced random-order blocks, but not in those who practiced fixed-order blocks. This improvement was anticipated for both tasks (task 1 and task 2) in the DT situation, and due to the higher-order nature of these skills, we expected these improvements to extend for both the trained tasks and untrained (transfer) tasks, demonstrating a task-unspecific enhancement.

On one hand, it seems reasonable to anticipate differences in the training outcomes between online and laboratory-based training regimens; for example, such disparities could arise from technical limitations (e.g., Elze and Tanner, 2012; Neath et al., 2011) especially due to testing on multiple days or potential decrease of participants’ motivation and attention over several days of online training (Jun et al., 2017; Kyewski and Krämer, 2018). However, we hypothesized no significant differences due to the advanced state of digital technology used for experimentation. Both, that is high measurement precision of current online platforms for experimentation (Anwyl-Irvine et al., 2021; Uittenhove et al., 2023) and the careful alignment of the experimental procedures between online and laboratory-based testing should allow for highly reliable and valid measurements even in experimental situations of psychological research aimed at testing higher level executive control functions (Gagné and Franzen, 2023; Sauter et al., 2020). Given the constrained interpretability of frequentist statistics, especially for null effects (e.g., van den Bergh et al., 2020; Wagenmakers et al., 2018b), we employed Bayesian model statistics in order to test for potentially lacking differences in the effects between the two settings. For that purpose, we tested various models, each with or without effects involving the training setting condition factor. If models lacking this factor demonstrated the most optimal fit to our data, it would imply that the setting of the training (laboratory vs. online) has no remarkable influence on the outcome of training and transfer effects.

## 2 Materials and methods

### 2.1 Participants

A total of 62 participants took part in the study, 28 in the laboratory setting and 34 in the online setting. We conducted a power analysis with MorePower 6.0 (Campbell and Thompson, 2012) for the laboratory setting condition assuming an effect sizes (partial eta square, *η*^2^_p_) of 0.32. This effect size was derived from previous training studies, demonstrating group-specific improved inter-task coordination (effects in dual-task costs) with effect sizes between 0.25 and 0.32 (Liepelt et al., 2011; Schubert and Strobach, 2018; Strobach et al., 2012a). It also matches the effect size found by Kübler et al. (2018) on an effect of an instruction manipulation on TOC costs (*η*^2^_p_ = 0.34). For detecting similar effect sizes for our relevant three-way interaction (as described below), the power analysis indicated that a group size of 14 participants per group would yield sufficient power (> 0.9) with a set alpha level of 0.05. To compensate for potential dropouts especially in the online setting, we deliberately increased the number of participants to 17 for each training order condition, for the online setting condition. Participants (75% female) were recruited from the Martin-Luther-University Halle-Wittenberg and had a mean age of 23.2 years (*SD* = 3.8). All participants had normal or corrected-to-normal vision and 95% were right-handed. Informed consent was collected from each participant. Participants were paid for participation at a rate of one course credit or seven euro per hour plus performance-based bonuses. Participants were assigned to one of two training order conditions (random DT training or fixed DT training) and to one of two setting conditions (laboratory or online). The first 12 participants per setting condition were randomly assigned to the two training order conditions after the pre-training session. All subsequent participants were assigned to a training order condition, so that the two conditions’ group size would remain as similar as possible on our critical depended variable, namely TOC costs (for a similar procedure see Jaeggi et al., 2014). Participants in the laboratory setting condition received a personal instruction and debriefing on each day they came to the laboratory, while in the online setting, these interactions were moved to an online video conference platform of the university,^1^ where the participant and the experimenter met at the start and at the end of each session, just like in the laboratory. The actual experiment itself was conducted on participants’ own computers in their homes. This approach allowed for parallel testing in the online setting, as only the instructions and debriefings needed to be scheduled; while one participant proceeded with the experiment, the next could receive their instruction. In contrast, parallel testing in our laboratory was not possible due to restricted laboratory space. Overall, despite our anticipation of potential dropouts (especially in the online setting), all participants completed every session, whether they were in the laboratory or online.

### 2.2 Apparatus and component tasks

The experiments were programmed in PsychoPy3 (Version 2021.1.4, Peirce et al., 2022). In the laboratory setting condition, participants sat in front of a 24-inch LCD monitor with a resolution of 1920 × 1,080 pixels and a refresh rate of 144 Hz. For the online setting condition, the experiment built in PsychoPy3 was hosted online on the platform Pavlovia^2^. Participants received instructions not to use a mobile device; however, they were allowed to use their computer equipped with a suitable keyboard at their homes. During pre-, training and post-session participants conducted different versions of two visual sensorimotor component tasks, to test for training effects (trained tasks) as well as for transfer effects (transfer tasks). Visual stimuli of the component tasks were presented centrally on the screen. The size of the stimuli did not scale with the size and resolution of participants’ screens at home, but remained of constant height and width for both laboratory and online settings. With the instruction to sit at a distance of approximately 75 cm, the stimuli of both visual component tasks were within the field of foveal vision of 2° visual angle and were presented in black font on a gray background.

Stimuli of the trained shape task consisted of the shapes circle, triangle and square, and for the transfer shape task, the shapes cross, star and hexagon were presented. Participants were instructed to respond as fast and as accurately as possible to the shape with the ring, middle or index finger of their left hand to press buttons ʻA,’ ʻS,’ or ‘D,’ respectively. For the trained symbol task, the letters ‘A,’ ‘E,’ and ‘U’ were used, while for the transfer symbol task, digits ‘1,’ ‘5,’ and ‘9’ were presented. Participants were instructed to respond as fast and as accurately as possible to the symbol with the index, middle or ring finger of their right hand to press buttons ʻJ,’ ʻK,’ or ‘L’, respectively.

A single task (ST) trial started with the presentation of a fixation cross for 800 ms, followed by a blank display for 700 ms. Then the visual stimulus appeared centrally on the screen and stayed until participants responded with a button press or until a maximum of 3,000 ms passed. Error feedback was given when the answer was incorrect as well as after omitted responses and consisted of the German words ‘FEHLER (error) and ‘ZU LANGSAM’ (too slow), respectively.

**Figure 1:**
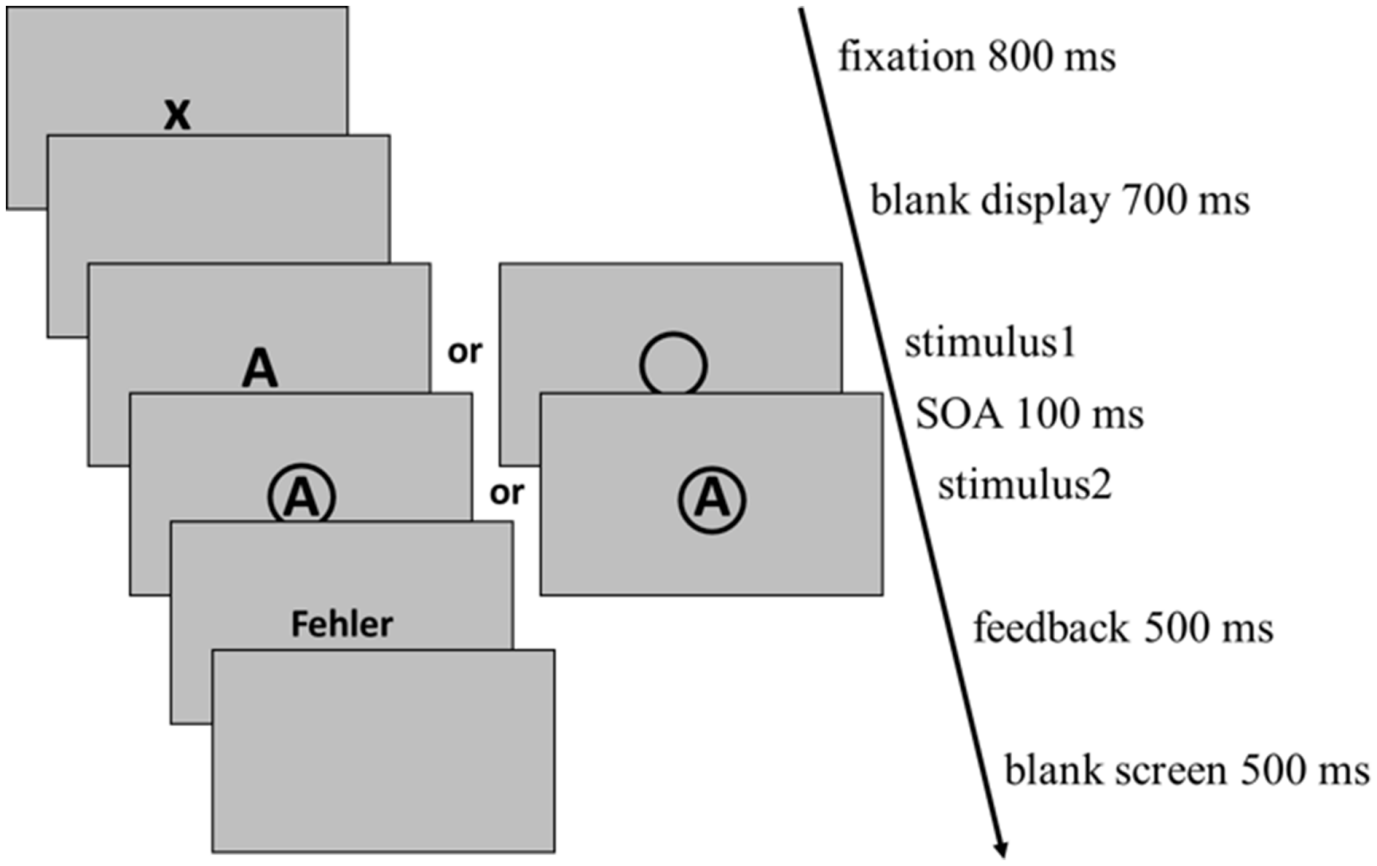
The time course of a DT trial as it was applied in fixed-order DT blocks as well as in random-order DT blocks. Following a fixation cross (800 ms) and a blank display (700 ms) both stimuli were presented separated by an SOA of 100 ms. The maximum time for both responses was set to 3,000 ms. Both stimuli remained on screen until both responses were given or until this maximum passed. After an ITI of 1,000 ms consisting of 500 ms error feedback (if there was an error) and 500 ms blank display the next trial started. ITI, intertrial interval; SOA, stimulus onset asynchrony.

A DT trial, again, started with the presentation of a fixation cross for 800 ms, followed by a blank display for 700 ms (see Figure 1). Then the first visual stimulus appeared centrally on the screen and with a stimulus onset asynchrony of 100 ms the second visual stimulus was presented. Both stimuli stayed on the screen until participants responded with a button press for each task or until a maximum of 3,000 ms after the appearance of the second stimulus passed. For DT blocks, participants were additionally instructed to respond according to the order of stimulus presentation. Error feedback was given when at least one stimulus discrimination was incorrect (but not if the order was incorrect) as well as after omitted responses and consisted of the German words ‘FEHLER (error) and ‘ZU LANGSAM’ (too slow), respectively.

### 2.3 Design and procedure

**Figure 2:**
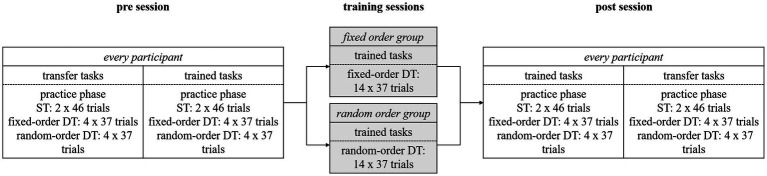
Overview for the procedure of blocks and trials for the different practice sessions of the study. Participants completed one pre session, three training sessions and one post session during the course of the study. Pre and post session: For the transfer tasks as well as for the trained tasks they performed two blocks of ST trials (one for the shape task, one for the symbol task), followed by four blocks of fixed-order trials (two for each task order), followed by four blocks of random-order trials. Training sessions: participants trained the two component tasks either in fixed-order DT blocks or in random-order DT blocks according to their training order condition assignment. ST, single task; DT, dual-task.

Participants engaged in the experiment over five consecutive days covering (if possible) one single week, as illustrated in Figure 2. Commencing on Monday, irrespective of their later training order condition assignment, participants started the study with a pre-session that lasted approximately 90 min. Initially, they practiced the transfer component tasks in nine single-task (ST) trials each to familiarize themselves with the mappings. Subsequently, they conducted two practice blocks involving the same component tasks within dual-task (DT) trials, featuring a random task order. Following the practice phase, participants completed 46 ST trials for each component task, followed by four blocks of 37 DT trials with fixed order. These fixed-order DT blocks encompassed two blocks for each possible order of component tasks in an alternating sequence (shape – symbol or symbol – shape). The order of stimulus presentation remained constant throughout each entire fixed-order block. Subsequently, participants conducted four blocks with 37 DT trials in random order of the component tasks. The number of trials per DT block was determined by the need to fully balance different experimental conditions: there were three possible stimuli for each task, two possible task orders and two possible order transitions from the previous trial, i.e., the order of the current trial could either repeat the previous order or be reversed. This balancing resulted in 36 trials (3 × 3 × 2 × 2) for each block, with one additional trial at the beginning (due to there was no transition from the previous one) which was excluded from analysis. For these random blocks, we pseudo-randomized the order of stimulus presentation before the start of the experiment. For the pseudo-randomization we adhered to the following rules: (1) the number of trials in which the order changed compared to the previous trial and trials in which the order repeated was equal; (2) within trials of order repetition, the number of both possible task orders (shape – symbol and symbol – shape) was equal; (3) within trials of order changes, the number of both possible task orders was equal; (4) the task order changed or repeated by a maximum of four consecutive trials.

After the random-order blocks with transfer component tasks, the procedure was repeated for the trained component tasks: a practice phase, 46 ST trials per component task, four blocks of 37 fixed-order DT trials and four blocks of random-order DT trials.

After the pre-session, participants were allocated to one of two training order conditions, as detailed earlier. On Tuesday, Wednesday and Thursday, participants completed one training session each. Participants in the fixed-order training condition practiced the two trained component tasks in 14 fixed-order DT blocks, each comprising 37 trials, with an alternating sequence of order between blocks. Conversely, participants in the random-order training condition engaged in 14 random-order DT blocks for the two trained component tasks, which were also pseudorandomized.

Finally, on Friday, all participants finished the study with a post-session, structured similarly to the pre session. However, in the post-session, the block involving the trained component tasks was administered before the block featuring the transfer component tasks. This sequencing ensured that the participants’ post-session performance in the trained tasks could *not* be influenced by the performance in the transfer tasks, because the latter were tested after the trained tasks.

### 2.4 Statistical procedure

For the statistical RT analyses, we excluded all trials from practice blocks and the initial trial of each DT block as well as trials with RTs deviating more than ±2.5 standard deviations from the mean of each specific factor combination for each participant (across laboratory and online setting *m* = 7.7%). Additionally, we removed error trials, encompassing both discrimination errors and those with incorrect task order (across laboratory and online setting *m* = 13.4%). RTs and error rates were aggregated across trials with the figure or the symbol task as task 1.

To investigate the acquisition of TOC skills, we compared DT performance, i.e., the RTs and error rates, in blocks with random task order with those in fixed task order. The RT and error rate differences between these two block types reflect the TOC costs and the observation of significantly larger RTs and/or error rates in random compared to fixed-order blocks would be consistent the assumption of the operation of additional control processes involved in coordinating the processing order in random-compared to fixed-order blocks. To test whether there are training-order specific training and transfer effects, we analyzed the TOC costs in form of pure RT or error data both before and after training using separate analyses of variance (ANOVAs). These 2 × 2 × 2 ANOVAs included the within-subjects factors *test time* (pre training, post training) and *trial type* (fixed-order, random-order) as well as the between-subjects factor *training order* (fixed DT training, random DT training), individually for the trained and transfer tasks. In a first step, we conducted these ANOVAs for the laboratory setting condition, and subsequently for the online setting condition.

Following that, we examined whether the training and transfer effects were modulated by the setting condition. For that purpose, we performed an additional 2 × 2 × 2 ANOVA with the three factors *test time* (within-subjects: pre training, post training), *training order* (between-subjects: fixed DT training, random DT training), and *setting* (between-subjects: laboratory, online). For the sake of simplicity, for this analysis, we used as a dependent variable, the TOC costs (i.e., the difference scores for random minus fixed order blocks, both for RTs and errors) in order to reduce the complexity of the further analyses because this allowed us to skip the factor *trial type*.

In addition, we conducted a Bayesian ANOVA using JASP, Version 0.18 (JASP Team, 2023), for comparison of the training-order specific training and transfer effects on TOC costs. For this Bayesian analysis, we, again, used the difference scores as the dependent variable. This decision was made to streamline the factors involved and, consequently, allowed us to minimize the number of potential models. This is important for the case of Bayesian analysis, because here, as the number of factors increases, a valid drawing of conclusions becomes progressively more uncertain (van den Bergh et al., 2020; Wagenmakers et al., 2018a). In particular, we calculated the posterior probabilities of a model not including any effect of the factor *setting* (main effect and interactions) and compared it to a model containing such effects. Note, the Bayes factor BF_01_ provides information which of these two models better fits the data, with values smaller than 1 suggesting support for the model without any effects and values greater than 1 providing evidence for a model incorporating these effects. A model without any effect of the factor *setting* would suggest that online training of task-coordination skills is as effective as laboratory-based training, whereas a model including such effects would indicate a difference between online and laboratory-based training (van den Bergh et al., 2020; Wagenmakers et al., 2018a,b).

## 3 Results

### 3.1 Laboratory setting

#### 3.1.1 RT analyses

##### 3.1.1.1 Trained tasks

**Figure 3:**
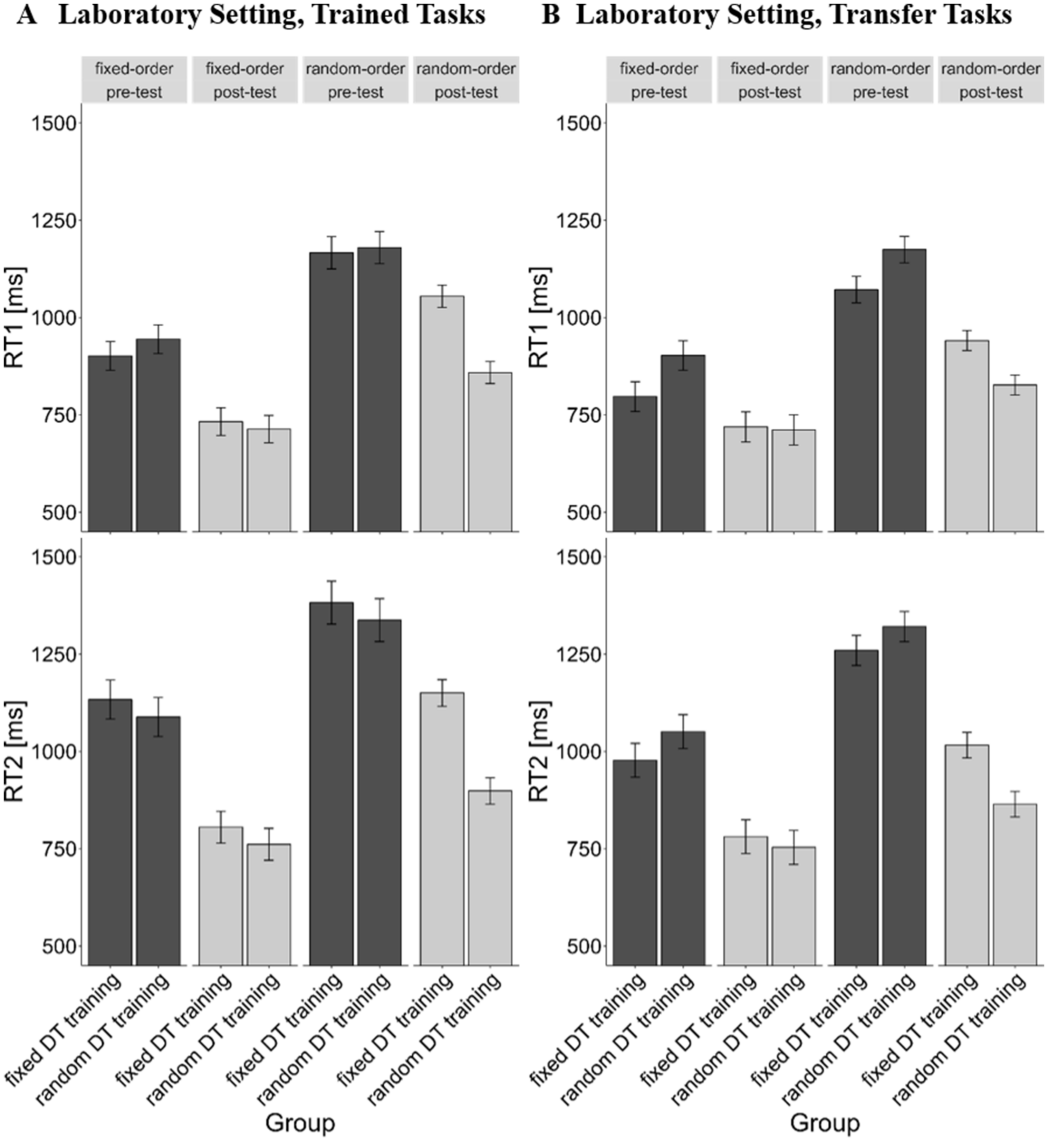
Mean RTs in ms as a function of test time (pre vs. post), trial type (fixed-order vs. random-order) and training order (fixed DT training vs. random DT training) for the laboratory setting. Error bars reflect the within-subject standard error of the mean (Cousineau, 2005). **(A)** RTs for the trained tasks of the laboratory setting. **(B)** RTs for the transfer tasks of the laboratory setting. *Fixed* = fixed-order trials, *random* = random-order trials, *pre* = pre training session, *post* = post training session.

First, we analyzed the RTs for the trained tasks (Figure 3A), focusing on reactions times for the first task presented in a trial (RT1). We observed significant main effects of the factors *test time*, *F*(1, 26) = 35.26, *p* < 0.001, *η*^2^_g_ = 0.17, and *trial type*, *F*(1, 26) = 162.10, *p* < 0.001, *η*^2^_g_ = 0.22. These findings indicate that RT1 was faster in the post-test (*m* = 879 ms) than in the pre-training session (*m* = 1,090 ms), signifying training effects, and, that responses in fixed-order blocks were executed more rapidly (*m* = 823 ms) than in random-order blocks (*m* = 1,065 ms), demonstrating the increased demand on TOC. Additionally, the *training order* × *test time* interaction depicts the tendency, *F*(1, 26) = 3.74, *p* = 0.064, *η*^2^_g_ = 0.02, that the reduction of RTs from pre-to post-test was more prominent in the random DT training condition (*mpre-minus _post-test_* = 291 ms; *p* < 0.001) than in the fixed DT training condition (*m_pre-minus post-test_* = 131 ms; *p* = 0.057). Furthermore, the *training order* × *trial type* interaction was significant, *F*(1, 26) = 7.35, *p* = 0.012, *η*^2^_g_ = 0.01, which reflects the fact that the TOC costs (difference of RT1 in random-order blocks minus RT1 in fixed-order blocks) of the random DT training condition (*m_difference_* = 190 ms) were smaller compared to those of the fixed DT training condition (*m_difference_* = 294 ms), *p* = 0.012. Most importantly, this interaction was furthermore moderated by the factor *test time* as indicated by the significant 3-way interaction *training order* × *test time* × *trial type*, *F*(1, 26) = 11.09, *p* = 0.003, *η*^2^_g_ = 0.01. While there was no difference in TOC costs between both *training order* conditions during the pre-test (*m_difference random DT training condition_* = 236 ms; *m_difference fixed DT training condition_* = 264 ms; *p* = 0.551), they differed during the post-test (*m_difference random DT training condition_* = 145 ms; *m_difference fixed DT training condition_* = 322 ms; *p* = 0.001). Training resulted in a reduction of TOC costs from pre-to post-test for the random DT training condition (*m_difference pre-minus post-test_* = 91 ms) but in an increase of TOC costs for the fixed DT training condition (*m_difference pre-minus post-test_* = −58 ms). This pattern is consistent with the assumption that training DT situations with random-order blocks improves TOC but training DT situations with fixed-order blocks does not.

The analysis of reaction times for the second task presented in a trial (RT2) replicated the outcomes observed for the RT1 analysis: It revealed significant main effects of *test time*, *F*(1, 26) = 29.75, *p* < 0.001, *η*^2^_g_ = 0.24, and of *trial type*, *F*(1, 26) = 175.98, *p* < 0.001, *η*^2^_g_ = 0.15, a significant *training order* × *trial type* interaction, *F*(1, 26) = 7.89, *p* = 0.009, *η*^2^_g_ = 0.01, as well as the significant 3-way interaction between *training order*, *trial type* and *test time*, *F*(1, 26) = 22.19, *p* < 0.001, *η*^2^_g_ = 0.01 on RT2. Similarly, in the random DT training condition, participants exhibited reduced TOC costs (*m_difference pre-minus post-test_* = 111 ms; *p* = 0.007) after training while participants in the fixed DT training condition demonstrated even an increase of the TOC costs (*m_difference pre-minus post-test_* = −95 ms; *p* = 0.018) after training.

##### 3.1.1.2 Transfer tasks

Regarding the transfer tasks (Figure 3B), for RT1, the main effects of *test time*, *F*(1, 26) = 34.33, *p* < 0.001, *η*^2^_g_ = 0.17, as well as *trial type* proved significant, *F*(1, 26) = 117.67, *p* < 0.001, *η*^2^_g_ = 0.22, mirroring the training effect (*m_pre_* = 1,032 ms; *m_post_* = 828 ms) and coordination demands (*m_random-order trials_* = 1,004 ms; *m_fixed-order trials_* = 783 ms) associated with the trained tasks. For the transfer tasks, we found a significant interaction between *training order* and *test time*, *F*(1, 26) = 6.68, *p* = 0.016, *η*^2^_g_ = 0.04, as well as between *test time* and *trial type*, *F*(1, 26) = 17.11, *p* < 0.001, *η*^2^_g_ = 0.12 on participants RTs. Participants in the random DT training condition exhibited a more pronounced reduction in RTs from pre-to post-test (*m_pre-minus post-test_* = 295 ms; *p* < 0.001) compared to the fixed DT training condition (*m_pre-minus post-test_* = 131 ms; *p* = 0.046). Furthermore, the RT reduction was more prominent in random-order trials (*m_pre-minus post-test_* = 239 ms; *p* < 0.001) than in fixed-order trials (*m_pre-minus post-test_* = 135 ms; *p* = 0.025). These findings were further specified by the 3-way interaction of *training order* × *test time* × *trial type*, *F*(1, 26) = 4.18, *p* = 0.051, *η*^2^_g_ < 0.01, which reflects the fact that, in the random DT training condition, participants improved TOC in random-order trials from pre-to post-test (*m* = 157 ms; *p* < 0.001), while in the fixed DT training condition, participants showed no improvement (*m* = 52 ms; *p* = 0.376). This result is in line with the hypothesis that training-related improvements of TOC, obtained during DT training with random-order blocks, also transfers to untrained tasks.

The analysis for RT2 provided similar results observed in RT1’s analysis, revealing two main effects: *test time*, *F*(1, 26) = 42.03, *p* < 0.001, *η*^2^_g_ = 0.28, and *trial type*, *F*(1, 26) = 119.78, *p* < 0.001, *η*^2^_g_ = 0.18. Additionally, the *test time* × *trial type* interaction reached significance, *F*(1, 26) = 15.08, *p* = 0.001, *η*^2^_g_ = 0.01, but the *training order* × *test time* interaction did not (*p* = 0.100). In addition, and most importantly, we found a significant 3-way interaction of *trial type*, *training order* and *test time* on RT2, *F*(1, 26) = 4.45, *p* = 0.045, *η*^2^_g_ < 0.01, which reflects the observation of a training-related reduction of TOC costs in the random DT training (*m_difference pre-minus post-test_* = 140 ms; *p* < 0.001), but no reduction in the fixed DT training condition (m_difference pre-minus post-test_ = 47 ms; *p* = 0.532).

#### 3.1.2 Error analyses

##### 3.1.2.1 Trained tasks

Subsequently, we examined error rates for both task 1 and task 2 (see Table 1). For the trained tasks, error rates for task 1 revealed a significant main effect of *trial type*, *F*(1, 26) = 22.46, *p* < 0.001, *η*^2^_g_ = 0.11, reflecting the occurrence of TOC costs at the error rate level, with higher error rates in random-order trials (*m* = 5.3%) compared to fixed-order trials (*m* = 3.0%). No other main effects or interactions reached significance. Similarly, in task 2, the main effect of *trial type* was also significant, *F*(1, 26) = 42.13, *p* < 0.001, *η*^2^_g_ = 0.09. Moreover, the main effect of *test time* reached significance, *F*(1, 26) = 6.62, *p* = 0.016, *η*^2^_g_ = 0.06, indicating a general reduction in error rates from pre-test (*m* = 5.6%) to post-test (*m* = 4.4%). None of the other main effects or interactions reached statistical significance.

**Table 1:** Error rates for task 1 and task 2 in percent (%) for the laboratory and the online setting.

	Laboratory setting				Online setting			
	Errors task 1		Errors task 2		Errors task 1		Errors task 2	
	Test time		Test time		Test time		Test time	
Training order condition/Tasks/Trials	Pre	Post	Pre	Post	Pre	Post	Pre	Post
**Fixed DT training**								
Trained tasks								
Fixed-order trials	3.2 (2.2)	2.1 (1.3)	5.4 (3.8)	2.7 (2.1)	5.3 (4.3)	4.0 (3.6)	6.4 (5.5)	3.2 (3.5)
Random-order trials	5.8 (4.0)	5.1 (3.1)	6.6 (4.7)	5.2 (3.9)	7.7 (6.2)	8.6 (7.4)	3.2 (3.5)	8.3 (7.4)
Transfer tasks								
Fixed-order trials	3.3 (2.9)	2.8 (3.5)	2.4 (1.9)	1.8 (1.4)	4.9 (6.4)	4.6 (5.7)	3.6 (3.1)	3.3 (2.0)
Random-order trials	5.0 (4.4)	5.1 (4.2)	3.1 (2.3)	2.4 (2.3)	10.0 (9.8)	7.9 (8.5)	5.6 (6.0)	6.9 (6.9)
**Random DT training**								
Trained tasks								
Fixed-order trials	3.9 (3.6)	2.9 (2.8)	4.2 (1.9)	3.2 (2.3)	3.3 (2.2)	1.9 (1.6)	4.2 (2.5)	2.6 (1.5)
Random-order trials	5.3 (3.6)	5.0 (6.5)	5.5 (3.0)	5.2 (3.6)	5.1 (3.7)	3.1 (3.2)	5.2 (3.3)	3.7 (3.0)
Transfer tasks								
Fixed-order trials	3.9 (2.7)	3.3 (2.8)	2.9 (1.7)	2.3 (2.8)	3.1 (2.9)	2.6 (1.8)	1.9 (1.1)	1.6 (1.5)
Random-order trials	5.3 (3.7)	5.9 (4.8)	4.7 (3.4)	3.8 (3.7)	4.6 (2.9)	3.6 (3.6)	2.9 (2.9)	2.3 (2.5)

##### 3.1.2.2 Transfer tasks

For the transfer tasks, we found a similar result pattern. We observed a main effect of *trial type*, for task 1, *F*(1, 26) = 21.96, *p* < 0.001, *η*^2^_g_ = 0.08, and for task 2, *F*(1, 26) = 10.23, *p* = 0.004, *η*^2^_g_ = 0.07, respectively, which indicates higher error rates in random-order trials (*m_task 1_* = 5.3%; *m_task 2_* = 3.5%) compared to fixed-order trials (*m_task 1_* = 3.3%; *m_task 2_* = 2.3%). Moreover, the main effect of *training order* was marginally significant, *F*(1, 26) = 3.34, *p* = 0.079, *η*^2^_g_ = 0.05 for task 2. No other main effects or interactions reached significance.

#### 3.1.3 Interim summary

Overall, the data of the laboratory setting condition revealed training-order specific training and transfer effects for TOC costs (on RT level), suggesting that participants can improve TOC through appropriate practice in random-order DT situations, which enable them to flexibly adjust the task order from trial to trial. Conversely, when participants practiced the same component tasks in fixed order, such improvements were not attainable. The findings were not contradicted by error rate analyses and no evidence for a speed-accuracy speed-off was observed.

### 3.2 Online setting

#### 3.2.1 RT analyses

##### 3.2.1.1 Trained tasks

**Figure 4:**
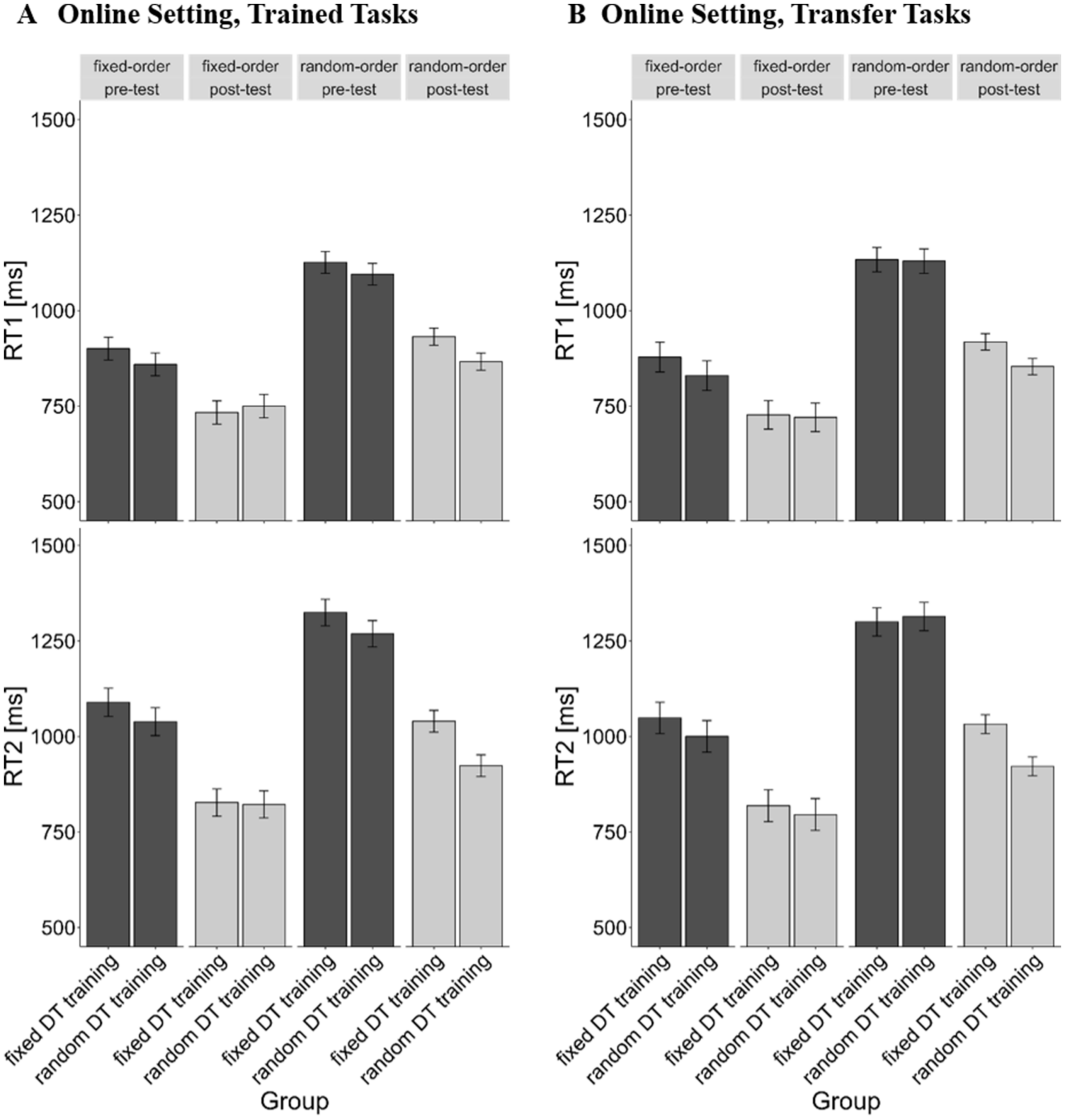
Mean RTs in ms as a function of test time (pre vs. post), trial type (fixed-order vs. random-order) and training order (fixed DT training vs. random DT training) for the online setting. Error bars reflect the within-subject standard error of the mean (Cousineau, 2005). **(A)** RTs for the trained tasks of the laboratory setting. **(B)** RTs for the transfer tasks of the laboratory setting. *Fixed* = fixed-order trials, *random* = random-order trials, *pre* = pre training session, *post* = post training session.

For the online setting, our findings closely paralleled those of the laboratory setting. First, we examined RT1 for the trained tasks (see Figure 4A), revealing significant main effects of *test time*, *F*(1, 30) = 50.98, *p* < 0.001, *η*^2^_g_ = 0.15, and *trial type*, *F*(1, 30) = 132.98, *p* < 0.001, *η*^2^_g_ = 0.17 on RT1. The former illustrates a general training effect, i.e., reduction of RTs from pre- (*m* = 1,034 ms) to post-test (*m* = 846 ms), while the latter depicts the typical demands of TOC, with higher RTs for random-order trials (*m* = 1,005 ms) compared to fixed-order trials (*m* = 811 ms). Additionally, the *test time* × *trial type* interaction reached significance, *F*(1, 30) = 14.11, *p* = 0.001, *η*^2^_g_ = 0.01, indicating that the reduction of RT1 from pre-to post-test was more pronounced in random-order trials (*m_pre-minus post-test_* = 212 ms; *p* < 0.001) than in fixed-order trials (*m_pre-minus post-test_* = 138 ms; *p* = 0.009). Most importantly, this interaction was furthermore moderated by the factor *training order*, as indicated by the significant 3-way interaction *training order* × *test time* × *trial type*, *F*(1, 30) = 5.58, *p* = 0.025, *η*^2^_g_ < 0.01. The random DT training group exhibited a reduction of TOC costs from pre-to post-test (*m_difference pre-minus post-test_* = 120 ms; *p* < 0.001), while this reduction was statistically not evident for the fixed DT training condition (*m_difference pre-minus post-test_* = 28 ms; *p* = 0.748). This finding aligns with the assumption that training DT situations with random-order blocks, but not with fixed-order blocks, enhances TOC, even an online setting.

The analysis for RT2 reproduced the patterns observed in RT1 analysis, revealing two significant main effects: *test time*, *F*(1, 30) = 52.10, *p* < 0.001, *η*^2^_g_ = 0.22, and *trial type*, *F*(1, 30) = 115.90, *p* < 0.001, *η*^2^_g_ = 0.12, as well as the *test time* × *trial type* interaction, *F*(1, 30) = 8.93, *p* = 0.006, *η*^2^_g_ = 0.01, and the 3-way interaction *training order* × *test time* × *trial type*, *F*(1, 30) = 4.38, *p* = 0.045, *η*^2^_g_ < 0.01. Similarly, participants in the random DT training condition showed decreased TOC costs after training (*m_difference pre-minus post-test_* = 129 ms; *p* = 0.004), while participants in the fixed DT training condition showed no enhancement due to training (*m_difference pre-minus post-test_* = 22 ms; *p* = 0.999).

##### 3.2.1.2 Transfer tasks

For RT1 of the transfer tasks (Figure 4B), we again found a significant effect of *test time*, *F*(1, 30) = 34.25, *p* < 0.001, *η*^2^_g_ = 0.16, and of *trial type*, *F*(1, 30) = 139.39, *p* < 0.001, *η*^2^_g_ = 0.20 on RT1. Duplicating results from the trained tasks, this signifies a general training effect (*m_pre_* = 1,039 ms; *m_post_* = 832 ms) as well as the typical demands of TOC (*m_random-order trials_* = 1,005 ms; *m_fixed-order trials_* = 811 ms) for the RT1 in the transfer tasks. Furthermore, the *test time* × *trial type* interaction proved significant, *F*(1, 30) = 17.65, *p* < 0.001, *η*^2^_g_ = 0.02, indicating a more prominent reduction of RT1 in random-order (*m_pre-minus post-test_* = 246 ms; *p* < 0.001) compared to fixed-order trials (*m_pre-minus post-test_* = 130 ms; *p* = 0.010). The 3-way interaction *training order* × *test time* × *trial type* was marginally significant, *F*(1, 30) = 3.46, *p* = 0.073, *η*^2^_g_ < 0.01, which reflects the fact that, in the random DT training condition, participants improved TOC with training (*m_difference pre-minus post-test_* = 166 ms; *p* < 0.001), while the training-related reduction of TOC costs was only minimal for the fixed DT training condition (*m_difference pre-minus post-test_* = 64 ms; *p* = 0.358). This observation supports the hypothesis that transfer effects resulting from training-related improvements of TOC can be realized in an online setting.

The analysis of RT2 paralleled again the results of RT1 analysis. Following the same pattern as with RT1, both main effects were significant: *test time*, *F*(1, 30) = 46.34, *p* < 0.001, *η*^2^_g_ = 0.22, and *trial type*, *F*(1, 30) = 131.45, *p* < 0.001, *η*^2^_g_ = 0.16, as well as the *test time* × *trial type* interaction, *F*(1, 30) = 11.623, *p* = 0.002, *η*^2^_g_ = 0.01. Most importantly, the 3-way interaction *training order* × *test time* × *trial type* reached significance, *F*(1, 30) = 4.97, *p* = 0.033, *η*^2^_g_ = 0.01, demonstrating a significant training-related TOC cost reduction for the random DT training condition (*m_difference pre-minus post-test_* = 187 ms; *p* < 0.001), but not for the fixed DT training condition (*m_difference pre-minus post-test_* = 38 ms; *p* = 0.994).

#### 3.2.2 Error analyses

##### 3.2.2.1 Trained tasks

Subsequently, we analyzed error rates for task 1 and task 2 (Table 1). Starting with the trained task 1, we observed a main effect of *trial type*, *F*(1, 30) = 17.44, *p* < 0.001, *η*^2^_g_ = 0.08, and a main effect of *training order*, *F*(1, 30) = 5.93, *p* = 0.021, *η*^2^_g_ = 0.12 on error rate in task 1. The former indicates the typical demands of TOC on error rate (*m_random-order trials_* = 6.1%; *m_fixed-order trials_* = 3.6%), while the latter depicts higher overall error rates for the random DT training condition (*m* = 7.0%) compared to the fixed DT training condition (*m* = 3.6%). Notably, the 3-way interaction *training order* × *test time* × *trial type* showed a pronounced trend, *F*(1, 30) = 3.86, *p* = 0.059, *η*^2^_g_ = 0.01, indicating that participants in the random DT training condition slightly improved TOC in terms of error rates in task 1 (difference of error rate in random-order blocks minus fixed-order blocks) from pre-to post-test (*m_difference pre-minus post-test_* = 0.6%), while participants in the fixed DT training condition demonstrated a decline in error-based performance (*m_difference pre-minus post-test_* = −2.2%). No other main effects or interactions reached significance.

For task 2, the main effect of *trial type* was significant, *F*(1, 30) = 15.27, *p* < 0.001, *η*^2^_g_ = 0.05, demonstrating higher error rates in random-order trials (*m* = 6.1%) compared to fixed-order trials (*m* = 4.1%). Furthermore, the significant main effect of *test time*, *F*(1, 30) = 15.77, *p* < 0.001, *η*^2^_g_ = 0.04, indicated a general reduction in error rates (*m_pre_* = 6.2%; *m_post_* = 4.7%). Moreover, the *training order* × *trial type* interaction was marginally significant, *F*(1, 30) = 3.86, *p* = 0.059, *η*^2^_g_ = 0.01, showing that the TOC costs in form of error rates differed between the training order conditions (*m_random DT training_* = 1.1%, *m_fixed DT training_* = 3.1%).

##### 3.2.2.2 Transfer tasks

Analysis of the transfer tasks produced a comparable outcome to that of the trained tasks. For task 1, the main effect of *trial type* was significant, *F*(1, 30) = 28.38, *p* < 0.001, *η*^2^_g_ = 0.06, which reflects the increased demands for TOC in random-order compared to fixed-order trials (*m_random-order trials_* = 6.5%; *m_fixed-order trials_* = 3.8%). The main effect of *training order* was marginally significant, *F*(1, 30) = 3.98, *p* = 0.055, *η*^2^_g_ = 0.09, with greater error rates for the fixed DT training condition (*m* = 7.6%) compared to the random DT training condition (*m* = 3.7%). Moreover, the significant *training order* × *trial type* interaction, *F*(1, 30) = 8.47, *p* = 0.007, *η*^2^_g_ = 0.02, indicated less pronounced TOC costs in form of error rates in the random DT training condition (*m* = 1.2%) compared to the fixed DT training condition (*m* = 4.3%).

For task 2, only the two main effects of *trial type*, *F*(1, 30) = 7.81, *p* = 0.009, *η*^2^_g_ = 0.06, and of *training order*, *F*(1, 30) = 7.31, *p* = 0.011, *η*^2^_g_ = 0.13, were significant, indicating higher error rates for task 2 in random-order trials (*m* = 6.5%) compared to fixed-order trials (*m* = 3.8%), as well as lower error rates for task 2 for the random DT training condition (*m* = 3.7%) compared to the fixed DT training condition (*m* = 7.6%). None of the other main effects or interactions reached statistical significance.

#### 3.2.3 Interim summary

In sum, data of the online setting condition yielded a result pattern analogous to that of the laboratory setting in terms of training-order specific training and transfer effects for TOC costs (on RT level). The error rate data again did not contradict these findings. For the online setting, participants in the random DT training condition was also able to enhance their TOC, independent of the specific stimuli used. In contrast, participants in the fixed DT training condition did not show such improvements.

### 3.3 Cross setting analyses

To further investigate whether the training and transfer effects observed in the online setting were comparable to those in the laboratory setting, we conducted an additional ANOVA including the factor *setting*. In order to reduce data complexity, we included a difference score reflecting the TOC costs across both tasks, i.e., task 1 and task 2, in the ANOVA. For that purpose, we collapsed the RTs for task 1 and task 2 together and calculated the difference between mean RTs in random-order trials minus mean RTs in fixed-order trials (i.e., RT_random-order_ – RT_fixed-order_) across both tasks. Using the resulting parameter as a dependent variable in the ANOVA allowed us to skip the factor *trial type* from the ANOVA and to calculate a 2 × 2 × 2 ANOVA with the factors *training order* (between-subjects: fixed DT training, random DT training), *test time* (within-subjects: pre, post), and *setting* (between-subjects: laboratory, online) on the TOC costs. We conducted the analysis separately for the trained and the transfer tasks. Notably, for both trained and transfer tasks, the interaction *training order* × *test time* was statistically significant, with *F*(1, 58) = 23.06, *p* < 0.001, *η*^2^_g_ = 0.101, for the trained task and with *F*(1, 58) = 9.24, *p* = 0.004, *η*^2^_g_ = 0.052, for the transfer task, respectively. This outcome confirmed the presence of training-order specific training and transfer effects as previously identified in analyses conducted separately for the two experimental settings. Most importantly, the factor setting did not significantly modulate these findings, as the interactions *training order* × *test time* × *setting* did not reach significance, for both, i.e., the trained tasks, *F*(1, 58) = 0.79, *p* = 0.378, *η*^2^_g_ = 0.004, and the transfer tasks, *F*(1, 58) = 0.07, *p* = 0.793, *η*^2^_g_ = 0.000, respectively. In sum, the training-order specific training and transfer effects remained unaffected of whether the study was conducted in laboratory or online setting.

#### 3.3.1 Bayesian analysis

Additionally, we conducted a Bayesian model comparison approach, in order to assess in more detail which model would best explain the current data set. In particular, we aimed to assess the observation of the former frequentist ANOVAs that the factor experimental setting has no reliable influence on the outcome of the training and transfer results. Please, note that in terms of a Bayesian analysis, this would be reflected by the observation of weak evidence for a model, which includes the factor *setting* compared to a model not including this factor. To assess this, we conducted two separate Bayesian repeated-measures ANOVAs, one for the TOC costs (difference values; RT_random-order_ – RT_fixed-order_ across task 1 and task 2) of the trained tasks and another for the TOC costs of the transfer tasks. All in all, a complete model testing would result in testing a total of 18 potential models against the best-fitting model, which are summarized in Appendix Table A1 for the trained tasks and in Appendix Table A2 for the transfer tasks. As the number of factors and models is very large in such complete model testing, it becomes increasingly uncertain to draw conclusions based on a comparison involving only a limited subset of these models (van den Bergh et al., 2020; Wagenmakers et al., 2018a). Thus, we performed Bayesian model averaging across matched candidate models, which are illustrated in Tables 2, 3 for the trained and transfer tasks, respectively. The aim is to preserve uncertainty in model selection by averaging the conclusions from each potential model, weighted by the model’s posterior plausibility (Wagenmakers et al., 2018a). The prior inclusion probability of an effect [i.e., the column P(incl)] aggregates the prior probabilities of all models containing that effect, while the posterior inclusion probability [i.e., the column P(incl|data)] represents the sum of posterior probabilities for those models. Crucially, the Bayes factor of inclusion (i.e., the column BF_incl_) indicates the change from prior to posterior inclusion odds, providing evidence for inclusion or exclusion of the respective effect (please, refer to Table 1 in Wagenmakers et al., 2018a for a descriptive classification scheme for the interpretation of Bayes factors).

**Table 2:** Analysis of effects for the trained tasks.

Effects	P(incl)	P(incl|data)	BF_incl_
Test time	0.263	5.0×10^−4^	3.938
Training order	0.263	6.7×10^−4^	14.440
Test time * training order	0.263	0.927	1268.191
Setting	0.263	0.205	1.248
Test time * setting	0.263	0.452	1.449
Group * setting	0.263	0.245	0.473
Test time * group * setting	0.053	0.072	0.517

*P(incl)* prior inclusion probability, *P(incl|data)* posterior inclusion probability, *BF_incl_* change from prior to posterior inclusion odds. Compares models that contain the effect to equivalent models stripped of the effect. Higher-order interactions are excluded. Analysis suggested by Sebastiaan Mathôt.

**Table 3:** Analysis of effects for the transfer tasks.

Effects	P(incl)	P(incl|data)	BF_incl_
Test time	0.263	2.6×10^−6^	54902.264
Training order	0.263	0.095	0.534
Test time * training order	0.263	0.056	15.119
Setting	0.263	0.677	0.259
Test time * setting	0.263	0.247	0.275
Group * setting	0.263	0.201	0.448
Test time * group * setting	0.053	0.017	0.463

*P(incl)* prior inclusion probability, *P(incl|data)* posterior inclusion probability, *BF_incl_* change from prior to posterior inclusion odds. Compares models that contain the effect to equivalent models stripped of the effect. Higher-order interactions are excluded. Analysis suggested by Sebastiaan Mathôt.

For the trained tasks (Table 2), the data supported the inclusion of the main factors *test time* (BF_incl_ = 3.938) with moderate evidence and *training order* (BF_incl_ = 14.440) with strong evidence. Notably, the interaction *test time* × *training order* received extreme support for inclusion (BF_incl_ = 1268.191). Most importantly, any model containing the factor *setting* received only anecdotal support for either inclusion or exclusion (all BF_incl_ within the range of 0.4–1.5). This confirms the conclusion that the way of investigating DT training, i.e., either in laboratory or in online setting, has no influence on the training performance of participants.

Turning to the transfer tasks (Table 3), the data overwhelmingly favored the inclusion of the main factor *test time* (BF_incl_ = 54902.264). Similarly as with the training tasks, the inclusion of the interaction *test time* × *training order* received strong evidence (BF_incl_ = 15.119). Again and most importantly for the purpose of the current study, any model involving the predictor *setting* received anecdotal or moderate support for exclusion (all BF_incl_ within the range of 0.2–0.5).

In summary, the Bayesian model comparison approach substantiated the conclusions drawn from classical inference analyses; it confirmed the observation of training-order specific training and transfer effects and, most importantly, it confirmed the conclusion that the training setting, i.e., whether or not training was conducted in laboratory or in online fashion, has no reliable impact on the training outcome.

## 4 Discussion

The primary objective of the current study was to investigate whether online training of dual-task performance can yield comparable results to those obtained in a laboratory setting. To this end, we conducted two distinct training regimens for dual-task situations across 5 days, an online and a laboratory environment, with two different training order conditions. Our aim was to evaluate the impact of training on dual-task costs, specifically focussing on the occurrence of TOC costs. These TOC costs represent the demands arising from the need to regulate the processing order due to bottleneck-induced sequential processing of two component tasks (De Jong, 1995; Kübler et al., 2022a,b; Liepelt et al., 2011; Szameitat et al., 2002). We aimed to investigate training effects and the subsequent stimulus-independent transfer of these effects, particularly in the context of TOC.

The results of the frequentist analyses of TOC costs revealed a reduction in TOC costs in participants who practiced two component tasks with a variable order, while those who practiced the same two tasks in a fixed order showed no such reduction from pre-to post-test session. Crucially, this training-related reduction in TOC costs generalized from the practiced to the transfer stimuli, which is indicative for the occurrence of stimulus-unspecific transfer effects of TOC skills. This finding suggests that the training influenced higher-order cognitive processes rather than mere consolidation of stimulus–response associations, which is in line with previous research demonstrating the development of generalizable task-coordination skills independent of specific task characteristics (Kramer et al., 1995; Liepelt et al., 2011; Schubert et al., 2017).

Moreover, the comparability of results between laboratory and online setting, evidenced by the absence of any influence of the factor *setting* in conventional frequentist statistics, emphasizes the robustness of this stimulus-unspecific training effects. Bayesian statistics further substantiated this conclusion by supporting the notion that the training setting did not affect training and transfer effects at all (van den Bergh et al., 2020; Wagenmakers et al., 2018a,b). Importantly, this highlights the resilience of the learning process to variations in training settings, whether conducted online or in a laboratory environment. Consequently, the benefits of the training regimen transcend the specifics of the training context, emphasizing its efficacy in fostering cognitive adaptation across diverse conditions.

The results of the study have important implications. First, it presents an extension of empirical evidence that practicing two component tasks with variable order can enhance TOC skills (Strobach, 2024), which goes beyond many previous findings observed by studies investigating DT training effects with varies training designs. In more detail, prior studies on DT training often employed DT situations in which both task stimuli were presented either simultaneously (e.g., Liepelt et al., 2011; Schubert et al., 2017; Strobach et al., 2015) or with a variable stimulus-onset asynchrony (e.g., Schubert and Strobach, 2018) and compared the resulting practice effects with practice effects of participants training only ST situations. While the studies attributed the observed advantage after training DT compared to training ST situations with the assumption that task-coordination skills had been acquired, it remained an open issue whether flexible TOC can be specifically optimized when training DTs requiring a flexible change and instantiation of different task orders. Our study extends the former findings by demonstrating that the acquisition of such task-*order* coordination skills can be promoted by appropriate dual-task practice. While participants practicing two component tasks in a fixed order did not enhance their TOC, those practicing these tasks in a variable order showed improvement of TOC in the trained tasks and, in addition, in unpracticed task situations.

Contrary to the findings of Strobach (2024), who observed training effects on TOC skills, our study focused on a different aspect of TOC. Strobach analyzed Order Switching (OS) costs, which refer to the difference in performance between same-order trials (where the order of task 1 and task 2 is repeated) and different-order trials (where the order is reversed) (see also Kübler et al., 2018). He found a reduction in OS costs following DT training with random order, but not after ST training. In contrast, we did not observe a similar group-specific improvement in our data (see Appendix B). However, we did find significant effects on TOC costs (measured through block-wise comparison) after DT training with random order, but not after DT training with fixed order. Strobach did not examine these TOC costs in his study. Given the notable differences between the two studies, further research is needed to fully understand TOC improvement.

This, in particular, indicates that various sub-mechanisms may have contributed to the observed training-related improvements; thus, the fact that participants in the random-order condition but not in the fixed-order condition improved TOC suggests that training has enhanced the monitoring of the stimulus order (Kübler et al., 2018) and, in addition, the instantiation and fast change of different task-order sets in working memory (Kübler et al., 2022b; Schubert et al., 2024; Schubert and Strobach, 2018). Both would have a positive impact on participants’ RT: enhanced monitoring enables a faster recognition of and therefore decision about the appropriate processing order; and a conjoint instantiation of different task-order sets leads to faster activation of the appropriate one in the current trial. However, further research is required, in order to disentangle mechanisms underlying improved TOC through practice.

The second major implication of this study pertains to the utilization of online assessments in cognitive training research. Our findings align with previous research indicating that data from online psychological experiments are comparable to those from equivalent laboratory-based experiments (e.g., Crump et al., 2013; Sauter et al., 2022; Semmelmann and Weigelt, 2017). We have shown that even when participants engage in a study involving complex tasks over several days, data remain consistent between laboratory and online settings. Effects that develop over time, as measured before and after the training regimen (training and transfer effects), were of similar magnitude and strength and were unaffected by the setting of the study. Consequently, we could provide robust effects for the improvement of TOC across diverse conditions, demonstrating independence from specific tasks (consistent effects for trained and transfer tasks) and settings (whether investigated online or in the laboratory). Hence, we advocate for online testing in cognitive training regimens, as such training would save tremendous resources without compromising quality of results.

A concern in cognitive training research pertains to the requisite number of training days for reliable effects. Previous studies often incorporated 8–15 days of practice (e.g., Liepelt et al., 2011; Ruthruff et al., 2006a,b; Schubert et al., 2017; Schubert and Strobach, 2018; Strobach et al., 2012b). However, these studies aimed to investigate transfer effects after extensive practice and skill automatization. In contrast, our research focused on the issue of online versus laboratory comparison of training effects. Therefore, a training regimen of 5 days including pre-and post-session seemed sufficient. To enhance the generalizability of the effects, future research may replicate these findings under various circumstances. Firstly, the impact of an extended training duration remains unclear. Potential effects of accumulated variances and diminishing motivation over the course of the training study may arise. Nevertheless, we demonstrated comparable effects between online and laboratory setting within a 5-day training regimen. Secondly, as our participants primarily consisted of young students, most of whom were psychology students, one could test a broader online sample. Lastly, our online setting was still very controlled, with an introduction and a debriefing via an online conference tool, mimicking a setting in the laboratory. Both, a more diverse sampling strategy as well as a more uncontrolled online setting can easily be achieved with the powerful tools available nowadays, e.g., Prolific or Amazon Mechanical Turk (Grootswagers, 2020; Sauter et al., 2020).

In summary, this study has demonstrated that stimulus-unspecific improvements in task(−order) coordination can be achieved by practicing two tasks in variable order. Importantly, these training and transfer effects were comparable when assessed in an online and in a laboratory setting. This supports the prospect of employing online assessments for cognitive training research inquiries, offering potential resources savings during data collection.

## Data Availability

The raw data supporting the conclusions of this article will be made available by the authors, without undue reservation.

## Footnotes

^1^mluconf.uni-halle.de/b

^2^https://pavlovia.org/
