# Supplementary material for: Comparing laboratory and online settings: equivalence in training and transfer effects for training task-order coordination processes
Source: Front Psychol. 2024 Oct 1;15:1440057. doi: 10.3389/fpsyg.2024.1440057 (PMC11473325; doi:10.3389/fpsyg.2024.1440057)
Supplement: Supplementary file 1 [file Table_1.docx]

# Appendix A

Table A1 Results of the complete of the Bayesian repeated measures ANOVA with all 18 potential models for the trained tasks

| Models | P(M) | P(M\|data) | BF_M_ | BF_10_ | error % |
| --- | --- | --- | --- | --- | --- |
| tt + gr + set + tt×gr + tt×set | .053 | .313 | 8.201 | 1.000 |  |
| tt+ gr + set + tt×gr | .053 | .205 | 4.645 | .665 | 4.508 |
| tt + gr + tt×gr | .053 | .164 | 3.538 | .525 | 3.700 |
| tt + gr + set + tt×gr + tt×set + gr×set | .053 | .139 | 2.895 | .443 | 4.010 |
| tt + gr + set + tt×gr + gr×set | .053 | .106 | 2.144 | .340 | 3.802 |
| tt + gr + set + tt×gr + tt×set + gr×set + tt×gr×set | .053 | .072 | 1.390 | .229 | 14.882 |
| tt + gr + set | .053 | 2.1×10^-4^ | 0.004 | 6.8×10^-4^ | 11.482 |
| tt + gr + set + tt×set | .053 | 1.8×10^-4^ | 0.003 | 5.8×10^-4^ | 4.325 |
| tt + gr | .053 | 1.8×10^-4^ | 0.003 | 5.8×10^-4^ | 8.198 |
| tt + gr + set + gr×set | .053 | 7.9×10^-5^ | 0.001 | 2.5×10^-4^ | 3.703 |
| tt + gr + set + tt×set + gr×set | .053 | 7.7×10^-5^ | 0.001 | 2.5×10^-4^ | 3.611 |
| gr + set | .053 | 5.1×10^-5^ | 9.1×10^-4^ | 1.6×10^-4^ | 3.304 |
| gr | .053 | 4.6×10^-5^ | 8.3×10^-4^ | 1.5×10^-5^ | 2.993 |
| gr + set + gr×set | .053 | 2.2×10^-5^ | 3.9×10^-4^ | 7.0×10^-5^ | 3.785 |
| tt | .053 | 1.3×10^-5^ | 2.4×10^-4^ | 4.3×10^-5^ | 2.876 |
| tt + set | .053 | 1.3×10^-5^ | 2.3×10^-4^ | 4.2×10^-5^ | 3.085 |
| tt + set + tt×set | .053 | 1.2×10^-5^ | 2.2×10^-4^ | 3.9×10^-5^ | 3.532 |
| null model (incl. subject and random slopes) | .053 | 4.0×10^-6^ | 7.2×10^-5^ | 1.3×10^-5^ | 2.703 |
| set | .053 | 3.8×10^-6^ | 6.9×10^-5^ | 1.2×10^-5^ | 3.374 |

*tt* test time, *gr* group, *set* setting, *P(M)* prior model probability, *P(M|data)* posterior model probability, *BF_M_* change from prior model odds to posterior model odds, *BF_10_* evidence for H_1_ vs. H_0_,

All models include subject, and random slopes for all repeated measures factors

| Models | P(M) | P(M\|data) | BF_M_ | BF_10_ | error % |
| --- | --- | --- | --- | --- | --- |
| tt + gr + tt×gr | .053 | .567 | 23.577 | 1.000 |  |
| tt + gr + set + tt×gr | .053 | .147 | 3.107 | .260 | 2.967 |
| tt | .053 | .071 | 1.382 | .126 | 2.003 |
| tt + gr + set + tt×gr + gr×set | .053 | .068 | 1.312 | .120 | 4.448 |
| tt + gr + set + tt×gr + tt×set | .053 | .042 | 0.789 | .074 | 3.896 |
| tt + gr | .053 | .039 | 0.721 | .068 | 3.143 |
| tt + set | .053 | .019 | 0.340 | .033 | 5.040 |
| tt + gr + set + tt×gr + tt×set + gr×set | .053 | .017 | 0.316 | .030 | 3.210 |
| tt + gr + set | .053 | .010 | 0.174 | .017 | 2.934 |
| tt + gr + set + tt×gr + tt×set + gr×set + tt×gr×set | .053 | .008 | 0.145 | .014 | 15.433 |
| tt + set + tt×set | .053 | .005 | 0.091 | .009 | 3.331 |
| tt + gr + set + gr×set | .053 | .004 | 0.072 | .007 | 2.802 |
| tt + gr + set + tt×set | .053 | .003 | 0.046 | .005 | 3.031 |
| tt + gr + set + tt×set + gr×set | .053 | .001 | 0.019 | .001 | 2.857 |
| null model (incl. subject and random slopes) | .053 | 1.4×10^-6^ | 2.4×10^-5^ | 4.3×10^-6^ | 1.697 |
| gr | .053 | 6.6×10^-7^ | 1.2×10^-5^ | 4.2×10^-6^ | 2.548 |
| set | .053 | 3.3×10^-7^ | 5.9×10^-6^ | 3.9×10^-7^ | 2.596 |
| gr + set | .053 | 1.5×10^-7^ | 2.7×10^-6^ | 1.3×10^-7^ | 6.716 |
| gr + set + gr×set | .053 | 6.0×10^-8^ | 1.1×10^-6^ | 1.2×10^-7^ | 6.193 |

*tt* test time, *gr* group, *set* setting, *P(M)* prior model probability, *P(M|data)* posterior model probability, *BF_M_* change from prior model odds to posterior model odds, *BF_10_* evidence for H_1_ vs. H_0_,

All models include subject, and random slopes for all repeated measures factors

Table A2 Results of the complete of the Bayesian repeated measures ANOVA with all 18 potential models for the transfer tasks
