# Supplementary material for: Comparing laboratory and online settings: equivalence in training and transfer effects for training task-order coordination processes
Source: Front Psychol. 2024 Oct 1;15:1440057. doi: 10.3389/fpsyg.2024.1440057 (PMC11473325; doi:10.3389/fpsyg.2024.1440057)
Supplement: Supplementary file 2 [file Table_2.docx]

# Appendix B

Table B1 Results of the 2 × 2 × 2 ANOVA for Order Switching costs on reaction time level for the laboratory setting condition on the first trained tasks (RT1)

| Predictor | *df_Num_* | *df_Den_* | *F* | *p* | η^2^_g_ |
| --- | --- | --- | --- | --- | --- |
| training order | 1 | 26 | 1.03 | .320 | .03 |
| test time | 1 | 26 | 34.25 | .000 | .16 |
| order transition | 1 | 26 | 20.58 | .000 | .01 |
| training order x test time | 1 | 26 | 8.02 | .009 | .04 |
| training order x order transition | 1 | 26 | 0.01 | .916 | .00 |
| test time x order transition | 1 | 26 | 2.64 | .116 | .00 |
| training order x test time x order transition | 1 | 26 | 0.00 | .982 | .00 |

| Predictor | *df_Num_* | *df_Den_* | *F* | *p* | η^2^_g_ |
| --- | --- | --- | --- | --- | --- |
| training order | 1 | 26 | 2.15 | .155 | .06 |
| test time | 1 | 26 | 29.84 | .000 | .23 |
| order transition | 1 | 26 | 17.68 | .000 | .01 |
| training order x test time | 1 | 26 | 2.83 | .105 | .03 |
| training order x order transition | 1 | 26 | 0.18 | .674 | .00 |
| test time x order transition | 1 | 26 | 2.47 | .128 | .00 |
| training order x test time x order transition | 1 | 26 | 0.86 | .363 | .00 |

*df_Num_* degrees of freedom numerator, *df_Den_* degrees of freedom denominator, *η^2^_g_* generalized eta-squared, *training order* factor training order (fixed DT training, random DT training), *test time* factor test time (pre training, post training), *order transition* factor order transition (same-order trials, different-order trials)

Table B2 Results of the 2 × 2 × 2 ANOVA for Order Switching costs on reaction time level for the laboratory setting condition on the second trained tasks (RT2)

*df_Num_* degrees of freedom numerator, *df_Den_* degrees of freedom denominator, *η^2^_g_* generalized eta-squared, *training order* factor training order (fixed DT training, random DT training), *test time* factor test time (pre training, post training), *order transition* factor order transition (same-order trials, different-order trials)

| Predictor | *df_Num_* | *df_Den_* | *F* | *p* | η^2^_g_ |
| --- | --- | --- | --- | --- | --- |
| training order | 1 | 26 | 0.00 | .946 | .00 |
| test time | 1 | 26 | 44.66 | .000 | .21 |
| order transition | 1 | 26 | 26.12 | .000 | .02 |
| training order x test time | 1 | 26 | 9.15 | .006 | .05 |
| training order x order transition | 1 | 26 | 0.15 | .706 | .00 |
| test time x order transition | 1 | 26 | 7.84 | .010 | .00 |
| training order x test time x order transition | 1 | 26 | 0.00 | .956 | .00 |

| Predictor | *df_Num_* | *df_Den_* | *F* | *p* | η^2^_g_ |
| --- | --- | --- | --- | --- | --- |
| training order | 1 | 26 | 0.26 | .616 | .01 |
| test time | 1 | 26 | 51.19 | .000 | .30 |
| order transition | 1 | 26 | 24.10 | .000 | .02 |
| training order x test time | 1 | 26 | 4.75 | .039 | .04 |
| training order x order transition | 1 | 26 | 0.13 | .722 | .00 |
| test time x order transition | 1 | 26 | 7.67 | .010 | .00 |
| training order x test time x order transition | 1 | 26 | 0.13 | .723 | .00 |

Table B4 Results of the 2 × 2 × 2 ANOVA for Order Switching costs on reaction time level for the laboratory setting condition on the second transfer tasks (RT2)

*df_Num_* degrees of freedom numerator, *df_Den_* degrees of freedom denominator, *η^2^_g_* generalized eta-squared, *training order* factor training order (fixed DT training, random DT training), *test time* factor test time (pre training, post training), *order transition* factor order transition (same-order trials, different-order trials)

*df_Num_* degrees of freedom numerator, *df_Den_* degrees of freedom denominator, *η^2^_g_* generalized eta-squared, *training order* factor training order (fixed DT training, random DT training), *test time* factor test time (pre training, post training), *order transition* factor order transition (same-order trials, different-order trials)

Table B3 Results of the 2 × 2 × 2 ANOVA for Order Switching costs on reaction time level for the laboratory setting condition on the first transfer tasks (RT1)

| Predictor | *df_Num_* | *df_Den_* | *F* | *p* | η^2^_g_ |
| --- | --- | --- | --- | --- | --- |
| training order | 1 | 32 | 0.38 | .540 | .01 |
| test time | 1 | 32 | 51.55 | .000 | .16 |
| order transition | 1 | 32 | 52.18 | .000 | .02 |
| training order x test time | 1 | 32 | 0.40 | .534 | .00 |
| training order x order transition | 1 | 32 | 1.32 | .260 | .00 |
| test time x order transition | 1 | 32 | 5.95 | .020 | .00 |
| training order x test time x order transition | 1 | 32 | 1.52 | .227 | .00 |

| Predictor | *df_Num_* | *df_Den_* | *F* | *p* | η^2^_g_ |
| --- | --- | --- | --- | --- | --- |
| training order | 1 | 32 | 1.06 | .312 | .03 |
| test time | 1 | 32 | 48.72 | .000 | .22 |
| order transition | 1 | 32 | 44.87 | .000 | .02 |
| training order x test time | 1 | 32 | 0.47 | .497 | .00 |
| training order x order transition | 1 | 32 | 3.20 | .083 | .00 |
| test time x order transition | 1 | 32 | 3.38 | .075 | .00 |
| training order x test time x order transition | 1 | 32 | 1.17 | .287 | .00 |

*df_Num_* degrees of freedom numerator, *df_Den_* degrees of freedom denominator, *η^2^_g_* generalized eta-squared, *training order* factor training order (fixed DT training, random DT training), *test time* factor test time (pre training, post training), *order transition* factor order transition (same-order trials, different-order trials)

Table B6 Results of the 2 × 2 × 2 ANOVA for Order Switching costs on reaction time level for the online setting condition on the second trained tasks (RT2)

*df_Num_* degrees of freedom numerator, *df_Den_* degrees of freedom denominator, *η^2^_g_* generalized eta-squared, *training order* factor training order (fixed DT training, random DT training), *test time* factor test time (pre training, post training), *order transition* factor order transition (same-order trials, different-order trials)

Table B5 Results of the 2 × 2 × 2 ANOVA for Order Switching costs on reaction time level for the online setting condition on the first trained tasks (RT1)

| Predictor | *df_Num_* | *df_Den_* | *F* | *p* | η^2^_g_ |
| --- | --- | --- | --- | --- | --- |
| training order | 1 | 32 | 0.15 | .698 | .00 |
| test time | 1 | 32 | 48.82 | .000 | .20 |
| order transition | 1 | 32 | 78.28 | .000 | .03 |
| training order x test time | 1 | 32 | 0.44 | .510 | .00 |
| training order x order transition | 1 | 32 | 1.40 | .246 | .00 |
| test time x order transition | 1 | 32 | 37.80 | .000 | .01 |
| training order x test time x order transition | 1 | 32 | 0.00 | .977 | .00 |

| Predictor | *df_Num_* | *df_Den_* | *F* | *p* | η^2^_g_ |
| --- | --- | --- | --- | --- | --- |
| training order | 1 | 32 | 0.35 | .559 | .01 |
| test time | 1 | 32 | 63.94 | .000 | .26 |
| order transition | 1 | 32 | 86.96 | .000 | .03 |
| training order x test time | 1 | 32 | 1.56 | .221 | .01 |
| training order x order transition | 1 | 32 | 2.21 | .147 | .00 |
| test time x order transition | 1 | 32 | 37.46 | .000 | .01 |
| training order x test time x order transition | 1 | 32 | 0.24 | .628 | .00 |

*df_Num_* degrees of freedom numerator, *df_Den_* degrees of freedom denominator, *η^2^_g_* generalized eta-squared, *training order* factor training order (fixed DT training, random DT training), *test time* factor test time (pre training, post training), *order transition* factor order transition (same-order trials, different-order trials)

Table B8 Results of the 2 × 2 × 2 ANOVA for Order Switching costs on reaction time level for the online setting condition on the second transfer tasks (RT2)

*df_Num_* degrees of freedom numerator, *df_Den_* degrees of freedom denominator, *η^2^_g_* generalized eta-squared, *training order* factor training order (fixed DT training, random DT training), *test time* factor test time (pre training, post training), *order transition* factor order transition (same-order trials, different-order trials)

Table B7 Results of the 2 × 2 × 2 ANOVA for Order Switching costs on reaction time level for the online setting condition on the first transfer tasks (RT1)
